# Accuracy of intraocular lens formulas using total keratometry in eyes with previous myopic laser refractive surgery

**DOI:** 10.1038/s41433-020-01159-5

**Published:** 2020-08-31

**Authors:** Tun Kuan Yeo, Wee Jin Heng, Don Pek, John Wong, Han Bor Fam

**Affiliations:** National Healthcare Group Eye Institute, Tan Tock Seng Hospital, Singapore, Singapore

**Keywords:** Outcomes research, Prognosis

## Abstract

**Objectives:**

This comparative study aimed to determine if total keratometry (TK) from IOLMaster 700 could be applied to conventional formulas to perform IOL power calculation in eyes with previous myopic laser refractive surgery, and to evaluate their accuracy with known post-laser refractive surgery formulas.

**Methods:**

Sixty-four eyes of 49 patients with previous myopic laser refractive surgery were evaluated 1 month after cataract surgery. A comparison of the prediction error was made between no clinical history post-laser refractive surgery formulas (Barrett True-K, Haigis-L, Shammas-PL) and conventional formulas (EVO, Haigis, Hoffer Q, Holladay I, and SRK/T) using TK values obtained with the optical biometer IOLMaster 700 (Carl Zeiss Meditec), as well as Barrett True-K with TK.

**Results:**

The mean prediction error was statistically different from zero for Barrett True-K, Barrett True-K with TK, Haigis-L, Shammas-PL, and Holladay I with TK. The mean absolute error (MAE) was 0.424, 0.671, 0.638, 0.439, 0.408, 0.424, 0.479, 0.647, and 0.524, and median absolute error (MedAE) was 0.388, 0.586, 0.605, 0.298, 0.294, 0.324, 0.333, 0.438, and 0.377 for Barrett True-K, Haigis-L, Shammas-PL, Barrett True-K TK, EVO with TK, Haigis with TK, Hoffer Q with TK, Holladay I with TK, and SRK/T with TK, respectively. EVO TK followed by Barrett True-K TK and Haigis TK achieved the highest percentages of patients with absolute prediction error within 0.50 and 1.00 D (68.75%, 92.19%, and 64.06%, 92.19%, respectively)

**Conclusions:**

Formulas combined with TK achieve similar or better results compared to existing no-history post-myopic laser refractive surgery formulas.

## Introduction

Intraocular lens (IOL) power calculation for cataract surgery in eyes with previous laser refractive surgery has been a challenge for many years [1]. Indeed, a great variety of formulas and algorithms have been described and validated for IOL power calculation in this type of eyes, with some requiring previous laser refractive surgery clinical history [2–6] and some without [2, 7–9]. No clinical history methods are popular due to their ease of use, the difficulty in patients obtaining their previous clinical history, and the variability in the accuracy of the clinical data. The Haigis-L [7] and Shammas-PL [8] formulas are examples of commonly used no-history methods. Recently, the Barrett True-K no-history formula has also been shown to be accurate [2, 10, 11].

Two of the main sources of errors in IOL power calculation in eyes with previous laser refractive surgery are the keratometric index error and effective lens position (ELP) error in formulas that utilize keratometry (K) to predict their ELP [7]. The use of standard K readings in conventional formulas results in overestimation of the corneal power and an erroneously anterior ELP, leading to hyperopic refractive surprises [12]. A potential solution to the keratometric index error would be the direct measurement of total corneal power.

One of the latest technologies for measurement of the total corneal power is the combination of a telecentric 3-zone K and swept-source optical coherence tomography technology from the IOLMaster 700 version 1.70 (Carl Zeiss Meditec AG, Jena, Germany) [13]. Specifically, this system provides a parameter called total keratometry (TK) which considers measured anterior and posterior corneal curvatures together with corneal thickness [14]. This may therefore be advantageous for patients whose anterior to posterior corneal relationships are altered, such as in post-laser in situ keratomileusis (LASIK) and keratoconus eyes [15]. Posterior corneal astigmatism has also been reported to be important in the implantation of toric IOLs [16]. Previously, total corneal power estimates have also been performed with measured anterior and posterior corneal curvatures and corneal thickness using Scheimpflug technology [9, 17].

The aims of this study were to determine if TK from IOLMaster 700 could be applied to conventional formulas to perform IOL power calculation in eyes with previous myopic laser refractive surgery, and to compare their results with known formulas specifically developed for this kind of eyes. It also aims to determine the accuracy of Barrett True-K with TK.

## Methods

### Patients

This comparative study enrolled eyes that underwent uneventful cataract surgery with previous laser corneal refractive surgery, either photorefractive keratectomy (PRK) or LASIK. All cataract surgeries were performed by four experienced surgeons at Tan Tock Seng Hospital, Singapore between 9 September 2017 and 8 March 2019. Inclusion criteria for the study were patients with uneventful cataract surgery, corrected distance visual acuity (CDVA) of 6/12 or better after cataract surgery, previous uneventful myopic PRK or LASIK, and successful biometric measurements with the IOLMaster 700 including TK (software version 1.70). Exclusion criteria included previous ocular surgery except PRK or LASIK, corneal pathology, retinal disorders or optic neuropathies with postoperative CDVA worse than 6/12, amblyopia, strabismus, and pupil abnormalities. This study conformed to ethics codes based on the tenets of the Declaration of Helsinki and was approved by the institution review board of the National Healthcare Group, Singapore.

### Clinical examination and surgical protocol

A complete pre-operative ophthalmological examination was performed in all cases, which included measurements of distance visual acuity, optical biometry (IOLMaster 700, Carl Zeiss Meditec AG, Germany), slit-lamp biomicroscopy, Goldmann applanation tonometry, corneal topography, and dilated fundoscopy. Cataract surgery was performed by four experienced surgeons (TKY, WJH, DP, HBF) using standard phacoemulsification through a temporal clear corneal incision. Postoperative pharmacological treatment consisted of a combination of antibiotic and steroidal anti-inflammatory drops. Patients were examined postoperatively at 1 day, 1 week, and 1 month after surgery. Manifest refraction was performed at the 1-month follow-up visit.

### Formula comparison

As the majority of patients did not have their previous refractive surgery history, comparison was made between the no-history post-refractive surgery formulas, namely the Barrett True-K (no history), Haigis-L, and Shammas-PL formulas, found on the American Society of Cataract and Refractive Surgeons (ASCRS) website, to conventional formulas not specifically developed for post-refractive surgery eyes, using TK values obtained with the IOLMaster 700, as well as the new Barrett True-K TK formula. The Barrett True-K TK formula, which utilizes measured posterior corneal power values from TK, is found on the Asia-Pacific Association of Cataract and Refractive Surgeons (APACRS) website. The conventional formulas were the Emmetropia Verifying Optical (EVO), Haigis [18], Hoffer Q [19], Holladay I [20], and SRK/T [21] formulas. The EVO formula is a new unpublished formula developed by the author (TKY) found at www.evoiolcalculator.com that has been recently compared favorably to other conventional formulas [22, 23].

As the Haigis formula does not use K or corneal power to predict its ELP, TK can be directly incorporated into the formula for its prediction. The EVO, Hoffer Q, Holladay I, and SRK/T formulas, however, all utilize K to predict their respective ELPs. Therefore, to avoid an error in ELP prediction, a novel “reverse double-K” method was applied using the equations shown below. (1) The mean posterior keratometry (PK) was converted to posterior radius. (2) Assuming that the posterior radius was not significantly altered by previous PRK or LASIK, the pre-refractive surgery anterior radius could then be calculated by dividing the measured posterior radius with the Gullstrand ratio of 0.883. This presumed pre-refractive surgery anterior radius obtained was then used to generate the ELP for each formula. (3) The measured TK value was converted to a composite radius based on the machine keratometric index. This is because IOLMaster 700 adjust TK values depending on the preset machine keratometric index (1.3375 for all eyes in this study) to ensure lens constants remain the same when using TK. The composite radius was then applied into each formula to generate their respective corneal powers.

(1) Pos *r* = (1376 − 1336)/PK = 40/PK

(2) Pre Ant *r* = −Pos *r*/0.883

(3) Comp *r* = 337.5/TK

Pos *r* = posterior radius

Pre Ant *r* = pre-refractive surgery anterior radius

Comp *r* = composite radius

TK = total keratometry

User Group for Laser Interference Biometry (ULIB) lens constants were used for the EVO, Haigis, Hoffer Q, Holladay I, and SRK/T formulas. The APACRS website recommended lens constants, if available, were used for the Barrett True-K and Barrett True-K TK formulas, otherwise the ULIB value was used (Table 1). Lens constant optimization was not performed for any of the formulas, to elicit any myopic or hyperopic bias. The predicted refraction was generated for each eye for each formula and deducted from the actual post-cataract surgery refraction to obtain the error in prediction. The mean error (ME) in prediction, mean absolute error (MAE), median absolute error (MedAE), standard deviation (SD), and percentages of eyes with absolute error <0.50, 0.75, and 1.00 D were then calculated. A box and whiskers plot of the prediction errors was also generated for each formula. For ease of reporting, all conventional formulas using TK were denoted with “TK” added to the formula name as a suffix (e.g., EVO TK = EVO using TK).

**Table 1:** Lens constants used.

	709M	AAB00	AR40e	ZCB00
Barrett True-K	118.5	119	118.71	119.39
EVO	118.5	119	118.7	119.3
Haigis	a0 0.637	a0 −1.004	a0 −2.420	a0 −1.302
	a1 0.400	a1 0.182	a1 0.157	a1 0.210
	a2 0.100	a2 0.232	a2 0.288	a2 0.251
Haigis-L	a0 0.637	a0 −1.004	a0 −2.420	a0 −1.302
	a1 0.400	a1 0.182	a1 0.157	a1 0.210
	a2 0.100	a2 0.232	a2 0.288	a2 0.251
Hoffer Q	5.37	5.56	5.41	5.80
Holladay I	1.59	1.78	1.63	2.02
Shammas-PL	118.5	119	118.7	119.3
SRK/T	118.5	119	118.7	119.3

### Statistical analysis

Data analysis was performed using statistical software SPSS version 26.0 (IBM, Armonk, NY, USA). The means of standard K and TK were compared using the two-tailed *t*-test. Normality of data samples was evaluated by means of the Shapiro–Wilk test. The one sample *t*-test was used to determine if there was any significant deviation from zero in the mean prediction error obtained with each formula. For pair-wise comparison of absolute errors, the nonparametric Friedman’s test with Bonferroni correction was used. A *p* value < 0.05 was considered to be statistically significant.

## Results

A total of 64 eyes of 49 patients with age ranging from 45 to 74 years old (average 56 years old) were included in the study. Three eyes (4.69%) had undergone previous PRK, whereas the remaining 61 eyes (95.31%) had undergone previous LASIK. The study sample included 28 (43.75%) male and 36 (56.25%) female eyes, and 34 (53.13%) and 30 (46.88%) right and left eyes, respectively. The following IOL models were implanted: AT TORBI 709M (*n* = 5; 7.81%) (Carl Zeiss Meditec, Jena, Germany), Sensar AAB00 (*n* = 37; 57.81%), Sensar AR40e (*n* = 3; 4.69%), and Tecnis ZCB00 (*n* = 19; 29.69%) (Johnson & Johnson Vision, Jacksonville, USA). The mean axial length, and IOL power implanted were 27.47 mm (range 24.46–32.49 mm) and 18.14 D (range 7.50–25.00 D), respectively. The mean standard K reading was 39.25 D (range 34.99–42.83 D), while the mean TK was lower at 38.15 D (range, 33.71–42.18 D). This difference was statistically significant (*p* < 0.001). Table 2 shows the patient demographics.

**Table 2:** Patient demographics.

	Mean ± SD	Range
Age (year)	56 ± 7	45–74
Axial length (mm)	27.47 ± 1.71	24.46–32.49
Mean standard keratometry (D)	39.25 ± 1.75	34.99–42.83
Mean TK (D)	38.15 ± 1.90	33.71–42.18
IOL power implanted (D)	18.14 ± 3.23	7.50–25.00

*TK* total keratometry.

**Fig. 1:**
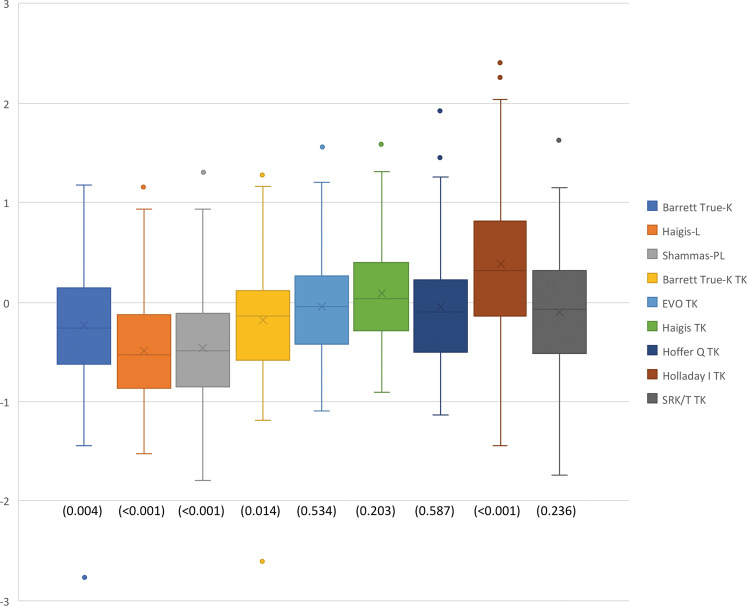
Box and whisker plots of prediction errors; no-history formulas followed by formulas using TK. *P* values from the one sample *t*-test used to determine significant difference from zero are presented for each formula.

The mean refraction prediction error was significantly different from zero for the Barrett True-K (*p* = 0.004), Barrett True-K TK (*p* = 0.014), Haigis-L (*p* < 0.001), Shammas-PL (*p* < 0.001), and Holladay I TK (*p* < 0.001) formulas. In contrast, the mean prediction errors obtained with EVO TK (*p* = 0.534), Haigis TK (*p* = 0.203), Hoffer Q TK (*p* = 0.587), and SRK/T TK (*p* = 0.236) were not significantly different from zero. The box and whisker plot of the prediction error for each formula is detailed in Fig. 1. The EVO TK formula had the lowest interquartile range (0.626), followed by Barrett True-K TK (0.643) and Haigis TK (0.661). The highest interquartile ranges were found for the Holladay I TK (0.948) and SRK/T TK (0.780) formulas. Comparing absolute error, statistically significant differences were found between the formulas (*p* < 0.001). Pair-wise post hoc analysis revealed that there were significant differences in absolute prediction error for the following comparisons: EVO TK vs. Haigis-L (*p* < 0.001), EVO TK vs. Holladay I TK (*p* < 0.001), EVO TK vs. Shammas-PL (*p* < 0.001), Barrett True-K TK vs. Haigis-L (*p* < 0.001), Barrett True-K TK vs. Holladay I TK (*p* = 0.012), Barrett True-K TK vs. Shammas-PL (*p* = 0.005), Haigis TK vs. Haigis-L (*p* < 0.001), Haigis TK vs. Holladay I TK (*p* = 0.005), Haigis TK vs. Shammas-PL (*p* = 0.002), Hoffer Q TK vs. Haigis-L (*p* = 0.014), and SRK/T TK vs. Haigis-L (*p* = 0.044). There was statistical difference between Barrett True-K against Haigis-L (*p* = 0.020) but not against Shammas-PL (*p* = 0.827) after Bonferroni correction.

**Fig. 2:**
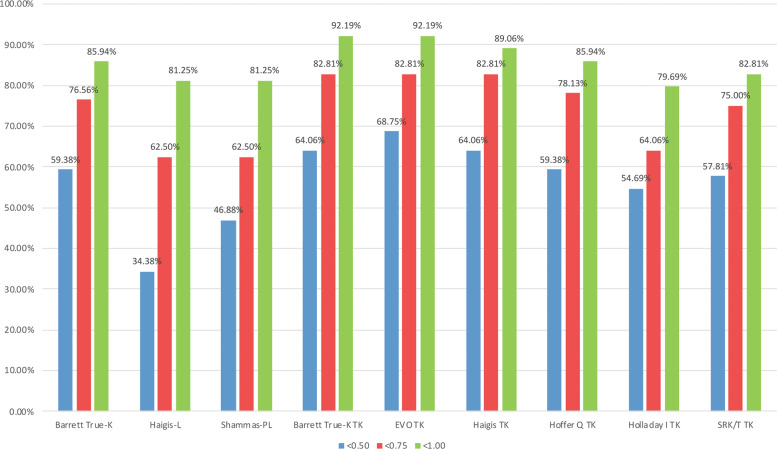
Percentage of eyes within 0.50 D, 0.75 D, and 1.00 D of absolute prediction error; no-history formulas followed by formulas using TK.

Table 3 details the mean numerical prediction error, SD of numerical error, MAE and MedAE for all formulas. The EVO TK formula had the lowest MAE (0.408), followed by Haigis TK (0.424), Barrett True-K TK (0.439), and Hoffer Q TK (0.479). The EVO TK formula also had the lowest MedAE (0.294), followed by Barrett True-K TK (0.298), Haigis TK (0.324), and Hoffer Q TK (0.333). For SD of prediction error, EVO TK was lowest at 0.535, followed by Haigis TK (0.554), Barrett True-K TK (0.590), and Shammas-PL (0.638). Figure 2 shows the percentage of eyes with absolute prediction error within 0.5, 0.75, and 1.00 D. The EVO TK formula had the highest percentage of eyes within 0.5 D error (68.75%). Its percentage of eyes within 0.75 and 1.00 D errors were 82.81 and 92.19%. This was followed by Barrett True-K TK (64.06, 82.81, 92.19%), Haigis TK (64.06, 82.81, 89.06%), Hoffer Q TK (59.38, 78.13, 85.94%), and Barrett True-K (59.38, 76.56, 85.94%).

**Table 3:** Predictive outcomes (laser refractive surgery formulas followed by formulas using TK in alphabetical order).

	Mean error ± SD	MAE	MedAE	Max error
Barrett True-K	−0.240 ± 0.645	0.512	0.388	2.765
Haigis-L	−0.490 ± 0.671	0.671	0.586	1.528
Shammas-PL	−0.461 ± 0.638	0.638	0.605	1.794
Barrett True-K TK	−0.186 ± 0.590	0.439	0.298	2.615
EVO TK	−0.042 ± 0.535	0.408	0.294	1.561
Haigis TK	0.089 ± 0.554	0.424	0.324	1.581
Hoffer Q TK	−0.043 ± 0.778	0.479	0.333	1.918
Holladay I TK	0.382 ± 0.778	0.647	0.438	2.414
SRK/T TK	−0.101 ± 0.674	0.524	0.377	1.745

*SD* standard deviation, *MAE* mean absolute error, *MedAE* median absolute error, *Max error* maximum absolute error.

Without the use of TK, the mean numerical prediction errors for EVO, Haigis, Hoffer Q, Holladay I, and SRK/T were all hyperopic: 0.996, 0.659, 0.754, 1.611, and 1.526, respectively. The MAEs were 1.005, 0.704, 0.786, 1.611, and 1.526, and the percentages of eyes with 0.5 D were 23.44, 51.56, 45.31, 6.25, and 6.25%.

## Discussion

The use of total corneal power for IOL power calculation is crucial in patients where standard K fails, due to the significant modification of the anterior to posterior curvature ratio of the cornea in eyes with previous laser corneal refractive surgery [7]. Currently, new biometers are introducing the concept of total corneal power for IOL power calculation, such as the TK parameter integrated in the IOLMaster 700 version 1.70 [13].

In our sample of eyes with previous laser refractive surgery, the mean prediction error was not significantly different from zero for EVO TK, Haigis TK, Hoffer Q TK, and SRK/T TK. The Barrett True-K TK, Barrett True-K, Haigis-L, and Shammas-PL formulas, on the other hand, were noted to have significant myopic tendency, and the Holladay I TK significant hyperopic tendency. Similar myopic mean prediction errors have been reported for the Barrett True-K no history [2], Haigis-L [2, 11, 24–26], and Shammas-PL [2, 11, 26] formulas. Although Holladay I TK performed well, its significant hyperopic ME may suggest it to be less suitable for post-myopic laser refractive surgery eyes in comparison to other conventional formulas using TK. The performance of EVO TK, Barrett True-K TK, and Haigis TK was particularly encouraging. EVO TK, Barrett True-K TK, and Haigis TK all performed better than Haigis-L, Holladay I TK, and Shammas-PL, while Barrett True-K, Hoffer Q TK, and SRK/T TK performed better than Haigis-L. The Barrett True-K no-history formula outcomes were similar to what was previously reported [2] (MAE: 0.512 vs. 0.520, MedAE: 0.388 vs. 0.410, SD: 0.645 vs. 0.670). Although current no-history formulas (Barrett True-K, Haigis-L, Shammas-PL) found on the ASCRS website have been shown to be accurate for IOL power calculation in eyes with previous corneal refractive surgery [10, 25–27], the use of formulas with TK may be a better option, using measured total corneal power rather than regression-based estimation. Indeed, prediction errors of similar or lower values have been reported with other approaches for post-LASIK eyes, using adjustments of corneal power with or without requirement of pre-laser refractive surgery data [4, 28–30]. Cho et al. [17] reported low prediction errors, comparable to those obtained with the Haigis-L and Barrett True-K formulas, with the use of the conventional Haigis formula and total corneal power measured with a Scheimpflug imaging-based device.

In this study, Haigis-L and Shammas-PL formulas, had the highest MAE with 34.38 and 46.88% of eyes having <0.50 D of absolute prediction error. In contrast, the EVO TK and Barrett True-K TK formulas achieved 68.75 and 64.06% of eyes with absolute prediction error of <0.50 D. Similarly, Wong et al. [24] found that the predictability of achieving a target within ±0.50 and ±1.00 D was 35.7% and 63.1%, respectively, when using the Haigis-L formula for IOL power calculation in eyes with previous myopic laser refractive surgery.

The use of a novel reverse double-K method as described above was validated with the results of the EVO TK, Hoffer TK, Holladay I TK, and SRK/T TK formulas. It is similar in concept to the Aramberri double-K method [5], but without the need for clinical history, instead using the PK value from TK to derive the pre-refractive surgery K. Therefore, the use of conventional formulas in combination with TK via a reverse double-K method, has been shown to be a valid option for calculating IOL power for eyes with previous myopic laser refractive surgery, comparable to or better than existing no-history methods. It would also be of interest to compare conventional formulas using TK to clinical history methods of IOL calculation for post-myopic laser refractive surgery eyes in the future. Further studies are also needed to confirm the utility of TK in eyes with previous laser refractive surgery for the correction of hyperopia and radial keratotomy, although some recommendations have been recently reported [24]. Mainly monofocal IOLs were studied in the majority of published studies on post-corneal refractive surgery IOL power calculations. Further research may include the assessment of TK formulas in eyes implanted with multifocal IOLs. A recent study has shown that the Shammas formula provided the least accuracy in predicting IOL power in eyes with multifocal IOL implantation after previous laser refractive surgery for myopia [11].

This study enrolled 64 eyes with previous corneal refractive surgery. This number is comparable or more than previous published studies [3, 4, 7, 8, 24]. Future studies of larger numbers would likely further support the findings of this initial pilot study. ULIB or website recommended lens constants were used rather than optimized constants for each IOL for each formula. This is because optimization should not be performed for post-refractive surgery formula comparisons as it would mask hyperopic or myopic tendencies of the formulas, and also does not represent the real clinical scenario where surgeons do not usually optimize lens constants for such patients. Four different types of IOLs were included in this study, each in fair numbers. Other similar studies have reported combinations of different IOL models [2, 23], because corneal refractive surgery patients are relatively uncommon.

In conclusion, the use of TK especially in conjunction with the Barrett True-K, EVO, and Haigis formulas is an accurate option for IOL power calculations in eyes with previous myopic laser refractive surgery.

## Summary

### What was known before

- IOL power calculation errors in eyes with previous refractive surgery include the keratometric index error and ELP error.

### What this study adds

- TK improves accuracy of the Barrett True-K and Haigis formulas, and together with the reverse double-K method enabled conventional formulas to predict for eyes with previous refractive surgery.
